# CORONAcrisis—An Observational Study on the Experience of Healthcare Professionals in a University Hospital during a Pandemic Emergency

**DOI:** 10.3390/ijerph18084250

**Published:** 2021-04-16

**Authors:** Teresa Matarazzo, Francesca Bravi, Giorgia Valpiani, Chiara Morotti, Francesca Martino, Sandra Bombardi, Michela Bozzolan, Elda Longhitano, Paola Bardasi, De Vogli Roberto, Tiziano Carradori

**Affiliations:** 1 Intensive Care Unit, Emergency Department, S. Anna University Hospital of Ferrara, 44124 Ferrara, Italy; t.matarazzo@ospfe.it; 2 Santa Maria Delle Croci Hospital, 48121 Ravenna, Italy; francesca.bravi@auslromagna.it; 3 Research Innovation Quality and Accreditation Unit, S. Anna University Hospital of Ferrara, 44124 Ferrara, Italy; chicca.morotti94@gmail.com (C.M.); s.bombardi@ospfe.it (S.B.); 4 Mental Health Center, Local Health Unit of Bologna, 40124 Bologna, Italy; francesca.martino2@ausl.bologna.it; 5 Training and Updating Unit, S. Anna University Hospital of Ferrara, 44121 Ferrara, Italy; bzh@unife.it; 6 General and Healthcare Administration, S. Anna University Hospital of Ferrara, 44124 Ferrara, Italy; segreteria.ds@ospfe.it (E.L.); segreteria.dg@ospfe.it (P.B.); 7 Human Rights Centre, Department of Social Psychology and Development, University of Padova, 35131 Padova, Italy; roberto.devogli@unipd.it; 8 Local Health Unit of Romagna, General Directorate, 48121 Ravenna, Italy; direzione.generale@auslromagna.it

**Keywords:** COVID-19 pandemic emergency, healthcare professionals, stress disorders, mental health, mixed-method sequential explanatory design

## Abstract

The Coronavirus disease 2019 (COVID-19) pandemic continues to affect millions worldwide and has posed unique challenges to healthcare professionals. Caring for patients during a pandemic may have negative impacts on their mental health. We describe the first part of a study using a mixed-method sequential explanatory design (QUANT→QUAL). This quantitative part examines the experiences of healthcare professionals during the pandemic in a University Hospital in Italy. We performed a cross-sectional hospital-based survey involving all healthcare professionals between 19 May 2020 and 3 June 2020. Perceived Stress Scale, Patient Health Questionnaire, and General Anxiety Disorder scores were calculated, in order to assess how the pandemic emergency changed the occupational and social habits of the healthcare professionals. The mean age of the 275 respondents was 47 years and females accounted for 80.2%. A total of 29.8% had a Perceived Stress Scale (PSS) score ≥25, 22.9% scored ≥15 on the Patient Health Questionnaire (PHQ-9) scale, and 17.1% scored ≥15 on the General Anxiety Disorder (GAD) scale. Stress symptoms were mostly manifested for interviewees over 55, females, those who live far from their family, those who have only one child, and those who had a qualification lower than high school or who had a medical specialization. Our findings show a relevant level of psychological distress, anxiety, and depression in up to 30% of the sample, highlighting a significant psychological burden in all professionals.

## 1. Introduction

Coronavirus disease 2019 (COVID-19) is a global public health emergency, which has caused over 100,000 deaths in Italy. At present, more than three million people are known to have been infected, but healthcare professionals have been particularly affected, both in terms of infection and mortality. There have been almost 560 deaths among physicians (https://portale.fnomceo.it/elenco-dei-medici-caduti-nel-corso-dellepidemia-di-covid-19/; accessed on 15 April 2021), and healthcare professionals account for 4.3% (https://www.epicentro.iss.it/en/coronavirus/sars-cov-2-dashboard; accessed on 21 January 2021) of cases, although they comprise less than 1% of the total population (https://www.saluteinternazionale.info/2020/03/salvare-gli-operatori-sanitari/; accessed on 12 March 2020). The overwhelming burden placed on the healthcare system has also produced mental health effects among healthcare workers. Providing care to others during the COVID-19 pandemic can lead to stress, anxiety, fear, and other strong emotions. How you cope with these emotions can affect your well-being, the care you give to others while doing your job, and the well-being of the people you care about outside of work. During this pandemic, recognizing what stress looks like, learning how to cope with it and how to build resilience, and knowing where to go to get help are crucial. Experiencing or witnessing life-threatening or traumatic events affects everyone differently. In some circumstances, this distress can be managed successfully, thus reducing the associated negative health and behavioral outcomes. In other cases, some people may experience clinically significant distress or impairment, such as acute stress disorder, post-traumatic stress disorder (PTSD), or secondary traumatic stress. Compassion, fatigue, and burnout may also result from chronic workplace stress and exposure to traumatic events during the COVID-19 pandemic [1]. As defined by the World Health Organization (WHO), in the context of emergency management—in which normal organizational procedures are suspended for the benefit of extraordinary measures, aimed at reducing the risk of incurring in a disaster—organizations should be able to show a certain resilience [2], expressed not only as promptness in the reconversion and reorganization of services, in order to respond to the new needs induced by the emergency situation (resilience in context), but also as a capacity of the human capital involved in emergency management to face, process, and overcome the trauma generated by it (individual resilience) [3]. In this respect, there already exist numerous recommendations [4,5] and studies in the literature that have explored the impacts of health emergencies on employees, in terms of immediate psychological response [6], stress mitigation [7,8], individual resilience skills, and coping strategies [9,10], and effects due to burnout [11,12]. From these studies, the experience of medical staff members responding to past epidemics has shown that the impact on their mental health has not only short-term but also long-term effects. In particular, healthcare professionals showed higher levels of psychological burden and distress, developing more severe symptoms of anxiety, depression, and post-traumatic stress disorder even three years later than the general population who had been quarantined or survived [13].

In general, the epidemic phenomenon is a historically known one, as well as the tools for managing trauma on operators in emergency situations, due, for example, to natural disasters such as earthquakes or floods [14,15]. In the specific case of this health emergency, the context in which these tools need to be adapted and/or rethought in response to this epidemic is new; this is the first epidemic to be declared a pandemic by the WHO after the publication of the guidelines “Pandemic influenza preparedness and response” [16]. Although the WHO has already published several documents on the impact of the COVID-19 emergency, particularly that on health professionals [17], and new specific studies investigating the factors associated with mental health outcomes in response to emergencies have just been published [18,19,20], the literature is still devoid of a framework that incorporates both organizational and individual factors contributing to the adjustment to a pandemic emergency [21]. From a methodological point of view, the studies available to date that have explored the impacts of the COVID-19 emergency on employees—in particular, on healthcare professionals involved on the front line—have focused on quantitative investigation techniques; for example, through online surveys [6,22]. Still unexplored, however, is the evidence on this specific issue that emerges from the use of “mixed” investigation techniques, which fall within the context of mixed-method research [23]. The combination of quantitative and qualitative approaches in the research design of a single study, which is typical of “mixed” methodologies, can offer a broader understanding of complex phenomena [24,25].

In this paper, we report the first (quantitative) part of a sequential explanatory mixed-method study (QUANT→QUAL). The specific objective of this first part is to describe the COVID-19 emergency experienced by the healthcare professionals of the University Hospital of Ferrara—a tertiary care hospital in the Emilia-Romagna Region, Northern Italy—with specific reference to its impact on mental conditions, stress, anxiety, depression, and post-traumatic disorders. The second (QUAL) part of the study, which is currently ongoing, will explain the qualitative results. The final aim of our mixed-method study is to promote tailored actions, based on the different characteristics and needs of people working in the hospital, to equitably support the resilience of the population under study.

## 2. Materials and Methods

A mixed-method sequential explanatory design was used to conduct this study, by collecting, analyzing, and integrating quantitative and qualitative data [23]. The mixed-method paradigm is based on the principles and logic of pragmatism. According to this paradigm, the mixed use of qualitative and quantitative approaches results in a better understanding of the problem [26]. This is the first part of a sequential explanatory mixed-method study (QUANT→QUAL) [23]. To briefly describe the whole study, the first quantitative part was a cross-sectional, hospital-based survey using a Computer-Assisted Web Interview (CAWI) [27] questionnaire (closed-ended questions), sent to the entire population of the University Hospital healthcare professionals between 19 May 2020 and 3 June 2020 (survey period). Participation was voluntary and without compensation. The self-administered questionnaire comprised 48 items. The first section investigated the professional characteristics of the subjects: area (health, administrative, or technical), professional role (doctor, doctor in training, nurse, nursing aid, midwife, radiology technicians, and so on), year of employment, and unit of work, among others. We assessed professional and personal experience during the COVID-19 emergency, examining how it changed the occupational and social habits of the healthcare professionals, with three validating tools: Perceived Stress Scale (PSS-10) [28], Patient Health Questionnaire (PHQ-9) [29], and General Anxiety Disorder (GAD-7) [30]. The last section investigated socio-demographic characteristics: gender, age, marital status, educational level, number of children, and home/family-based distance. The questionnaire closed with an open question, where professionals could discuss the most considerable aspects of their experience at work during the pandemic emergency. We describe the methods of the first part in detail in this paper. The second (qualitative) part of the study intends to explain the results of the first (quantitative) part and to explore the new emerging themes in more depth, if the qualitative data suggest it. A purposeful sample from the same population who received the questionnaire will be selected. Statistically significant outcome results, key predictors, and distinctive demographic characteristics of the quantitative part will inform the selection of the qualitative sample and the topics of semi-structured individual interviews. Referring to a phenomenological approach [31], the small initial sample will be enlarged until the saturation of themes explaining the quantitative results and in-depth exploration of the new emerging concepts is ensured. The quantitative and qualitative integrated data will be interpreted and presented in a joint display, in order to gain a deeper comprehension of the phenomenon of interest and to identify initiatives to enhance the well-being of the considered professionals, during or after the pandemic.

The study protocol was approved by the Independent Ethical Committee of Area Vasta Emilia Centrale (CE-AVEC, study code: 503/2020/Oss/AOUFe; date of approval CE: 5 May 2020) and the study had administrative permission by the General Direction of Ferrara University Hospital.

### 2.1. Participants

During the survey period, all healthcare professionals received a hyperlink by email inviting them to take the survey. Subjects over the age of 18 who gave authorization of study participation were considered eligible. In the analysis, we included respondents who gave their informed consent to participate in this study and authorization to process personal data. No randomized sampling of the study population was performed.

### 2.2. Mental Health Outcomes

We focused on symptoms of stress, depression, and anxiety for all participants, using the PSS-10, PHQ-9, and GAD-7 scales (Italian versions), respectively. The PSS-10 scale is commonly used to evaluate people’s perception of particular situations in their daily life and their reaction in response to these events, perceived as destabilizing and risky. This questionnaire consists of 10 questions, with a value ranging from 1 to 5, depending on the severity. The total score is given by the sum of the scores attributed to each single question [28]. To identify people with more stress symptoms, we used a quartile split to separate the higher quartile from the remaining participants at ≥25 [32]. The PHQ-9 is a self-reported measure scale used for the diagnosis, monitoring, and determination of the severity of depression. The total score of the responses suggests varying levels of depression, categorized as follows: Minimal/no depression (0–4), mild depression (5–9), moderate depression (10–14), or severe depression (15–27) [18,22]. GAD-7 has seven items, which measure the severity of various signs of anxiety, according to the reported response categories, with the assigned points. The assessment is indicated by the total score, which is formed by adding together the scores for the scales of all seven items. The total scores are categorized as follows: Minimal/no anxiety (0–4), mild anxiety (5–9), moderate anxiety (10–14), or severe anxiety (15–21) [18,22]. The considered cutoff score for detecting symptoms of depression and anxiety for both PHQ-9 and GAD-7 scales was 15. Participants who had scores greater than the cutoff threshold were characterized as having severe symptoms.

### 2.3. Statistical Analysis

The Shapiro–Wilk test was used to test for normality of the distribution of age (continuous variable). In the presence of symmetry of the distributions, the variable is represented with the mean and standard deviation, and comparisons were assessed using Student’s *t*-tests. In the case of a nonnormal distribution, the variables are represented with the median value and interquartile range (IQR), and comparisons were assessed using the Wilcoxon–Mann–Whitney test for two independent samples. Categorical data (gender, age, education level, marital status, number of children, distance from family, and type of employment in the hospital) are expressed as total numbers and percentages. Statistical comparisons of categorical variables were assessed using Pearson’s *χ*^2^ test or Fisher’s exact test, depending on the minimal expected count in each crosstab. The relationships between PPS, PHQ, GAD scale, socio-demographic characteristics, and type of employment in the hospital (health, administrative, or technical) were assessed by the *χ*^2^ test. A multivariate logistic regression analysis was performed to estimate the Odds Ratios (ORs) for the total sample and stratified by gender. All analyses were performed using the Stata 14.1 SE software (Stata Corporation, College Station, TX, USA). A two-sided *p*-value < 0.05 was defined as statistically significant.

## 3. Results

**Figure 1:**
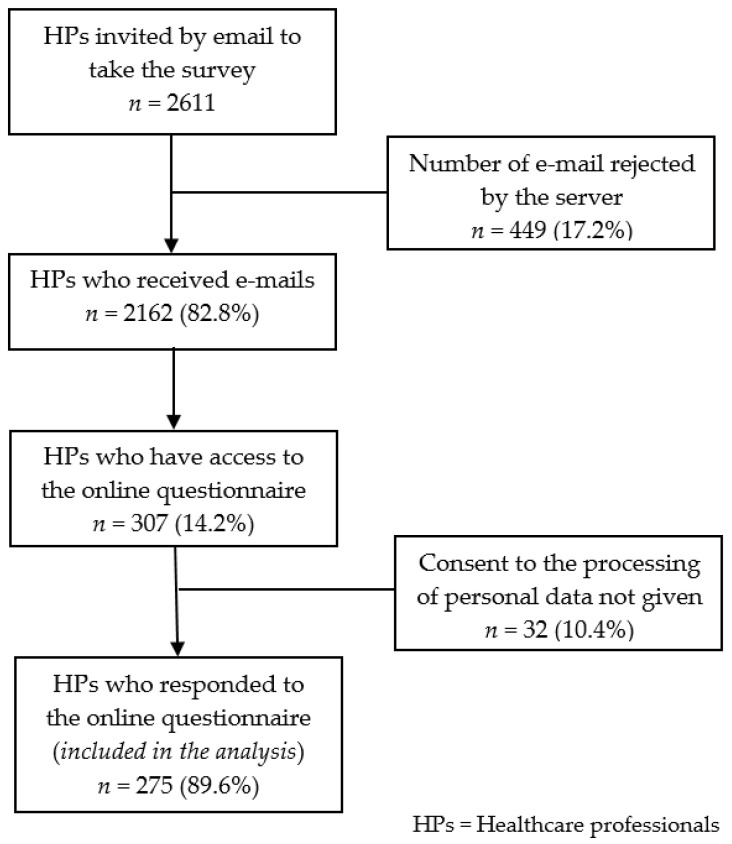
Flow chart.

During the survey period, three hundred and seven questionnaires were completed and two hundred and seventy-five questionnaires were considered in the analysis (32 were excluded because they had not given consent to the handling of data). Between 19 May 2020 and 3 June 2020, the response rate was 14.2% (Figure 1).

A description of the sample is provided in Table 1. The mean age of healthcare professionals who responded to the survey was 47 years (±10 years) and females accounted for 80.2%. The majority of the sample were healthcare professionals (89.5%) and most declared to be married (50.5%) or cohabitants (18.3%). A total of 174 respondents (63.3%) had a child and, of these 174, the majority (50%) declared having only one child. Of the 275 respondents, 181 (65.8%) lived close to their family.

Of the 275 respondents, 29.8% had a PSS score ≥25, 22.9% showed a value ≥15 on the PHQ-9 scale, and 17.1% scored ≥15 on the GAD scale. As reported in Table 2, the symptoms of stress were mostly manifested for female interviewers (*p* = 0.022), in people who lived far from their family (*p* = 0.013), for those who had only one child (*p* = 0.018), and for those who had a qualification lower than high school (*p* = 0.007); meanwhile, the proportion of respondents over 55 years old (*p* = 0.026) and the proportion of respondents with a medical specialization (*p* = 0.028) achieved PSS scores below the cut-off value (≥25). Scores ≥15 for the PHQ scale were associated only who having graduated high school (*p* = 0.007). Scores ≥15 for the GAD scale, on the other hand, were not associated with any factor present in Table 2. Multivariate logistic regression models were used to investigate possible predictors for stress, depression, and anxiety (Table 3). Considering the presence of stress symptoms, being older was a protective factor; in particular, the probability of being stressed decreased significantly by 57% for the 45–54 age class (OR = 0.43, Confidence Interval (CI) 95%: 0.19–0.99) and by 77% for the ≥55 age class (OR = 0.23, CI 95%: 0.09–0.59). Similarly, educational level was significantly associated with stress symptoms: having a bachelor’s or master’s degree or medical specialization decreased the probability of having stress symptoms by 75% (OR = 0.25, CI 95%: 0.12–0.53). Conversely, living far from family was associated with a twofold increased risk of stress (OR = 2.24, CI 95%: 1.18–4.20), as well as having only one child (OR = 3.05, CI 95%: 1.40–6.64; threefold increased risk of stress). Predictors of depression were investigated using the PHQ-9 model. In this case, a decreased risk of probability (by 66%) was found among people who had a bachelor’s or master’s degree or medical specialization (OR = 0.34, CI 95%: 0.16–0.71), as in the model used to investigate possible predictors for stress. No significant associations were found among variables and anxiety symptoms in the GAD-7 model. Stratifying by gender, as shown in Table 4, the predictors associated with symptoms of stress and depression were the same as those identified in Table 3 only for females. In fact, in the PSS model, being over 55 was a protective factor; thus, the probability of being stressed decreased by 73% (OR = 0.27, CI 95%: 0.09–0.73), while the probability decreased by 71% for those having a bachelor’s or master’s degree or medical specialization (OR = 0.29, CI 95%: 0.14–0.63). Having a child and living far from family were significant risk factors for stress symptoms; the likelihood of having stress symptoms increased more than twofold for women with a child and about twofold for those who lived far from family (OR = 2.82, CI 95%: 1.24–6.40; OR = 1.99, CI 95%: 1.01–3.91). In the model that identified possible predictors associated with depression symptoms, estimated only for females, having a bachelor’s or master’s degree or medical specialization decreased the probability of having depression symptoms by 73% (OR = 0.27, CI 95%: 0.12–0.63), as in the model in Table 3. No variables were found to be statistically significant for the models estimated for the male gender.

## 4. Discussion

The experiences of medical staff members responding to past epidemics (e.g., SARS, MERS, and Ebola) have demonstrated that the impact on their mental health has not only short-term but also long-term effects [7,33,34,35,36,37,38]. From previous research, healthcare professionals have shown higher levels of psychological burden and distress, and they developed more severe symptoms of anxiety, depression, and post-traumatic stress disorder, even three years later than the general population who had been quarantined or survived.

Recent studies [18,22,38,39] evaluating the mental health impact of the current COVID-19 pandemic have reported higher levels of depression, anxiety, and distress in healthcare professionals, compared to the general population, addressing their particular risk of developing acute stress disorders, especially if they were front-line workers. In our study, we assessed the psychological impact of the current pandemic on participants who work at Ferrara University Hospital (with both clinical and nonclinical roles). Our findings showed a relevant level of psychological distress, anxiety, and depression in up to 30% of the sample (independently if they were clinicians or had other roles), highlighting a significant psychological burden in all professionals who worked at Ferrara University Hospital, regardless of their specific working category; however, medical clinicians (doctors and nurses) composed the majority of sample (90%). Thus, this data should be replicated in further studies with bigger samples, in order to explore different psychological impacts on healthcare professionals with clinical or nonclinical roles. In our sample, we found slightly lower levels of psychological burden, compared to other studies [18,22,38,39] that have assessed the rates of distress, anxiety, and depression (ranging from 23–70%) in healthcare professionals during the COVID-19 pandemic. It is important to note that these studies were conducted in China, and most of the participants were doctors, nurses, and front-line workers in hospitals sited in the most impacted areas (Wuhan, other regions in Hubei province, and regions outside Wuhan province). Furthermore, our findings showed that distress symptoms were more severe in women, younger participants (<45 years), those with lower academic titles, and those who lived far from family. This is in line with the findings of other authors [18], who found a greater psychological burden in front-line female nurses working in Wuhan with junior titles and with fewer years of work experience. In conclusion, our findings highlight the need to immediately promote the mental health well-being of healthcare professionals exposed to COVID-19. Efficient and comprehensive actions should be taken as soon as possible, in order to protect the mental health of medical staff, especially those who have experienced more severe stress symptoms. In addition to that, periodical psychological screening tools should be adopted, in order to assess and monitor the mental health status of workers and to promptly recognize those who show signs of acute stress. In the literature, some authors [22] have investigated the subjective experience of healthcare professionals and have shown their great need to receive psychological support, in order to increase their self-help skills to face the pandemic burden and to help alleviate the psychological distress of others. Furthermore, they provided data on the first implementation of psychological interventions for healthcare professionals, with benefits in alleviating acute mental health disturbances and improving physical health perceptions. In order to define appropriate and effective interventions for this high-risk population, qualitative data are needed, in order to explore their subjective experience and to deeper-understand the emotional impacts, their psychological needs and difficulties, and the personal skills they have used to face the pandemic. Psychological needs in healthcare professionals may be different, compared to the general population. They may experience fear and anxiety related to being infected and infecting their relatives, feelings of helplessness in facing the pandemic and in helping people, stigmatization and social and behavioral avoidance, and an increasing burden of work with less time to rest. In order to address the subjective experience of the COVID-19 pandemic on healthcare professionals, we will implement the second part of the present research through a qualitative approach that explains the results of the first. Since the advent of the COVID-19 pandemic, the Emilia Romagna Region has provided specific psychological support for different population groups (e.g., the general population, people who have been infected, relatives of infected or dead people, and healthcare workers), in line with WHO recommendations (WHO 2020) [4]. The psychological intervention was on-line or provided by telephone, and aimed to: (1) spread appropriate information about the pandemic; (2) disseminate coping strategies to deal with stress; and (3) support people with distress symptoms, mild anxiety, and depression through psychological counselling. For more severe psychiatric symptoms, specialist assessments and interventions were provided by local Community Mental Health Centers. Collecting qualitative data could help psychologists to develop improved clinical strategies to provide different interventions, depending on risk populations and their needs. Authors should discuss the results and how they can be interpreted from the perspective of previous studies and of the working hypotheses. The findings and their implications should be discussed in the broadest context possible. Future research directions may also be highlighted.

The limitations of this study include a lack of baseline data on stress, anxiety, and depression levels before the pandemic period. A nonresponse bias may have also affected the results, given the low response rate in our study sample [40]; however, it should be interpreted considering the pandemic and the brief period (19 May 2020 to 3 June 2020) in which the survey was conducted. It is possible that nonrespondents may differ, in a systematic way, compared to respondents. The results of this study cannot be conclusive, as this quantitative part is the first of the series and information from the qualitative study may possibly make up for the low response rate.

## 5. Conclusions

Mental health challenges have been experienced by healthcare workers during the current COVID-19 pandemic, showing that there are high risks of developing psychiatric symptoms or diseases. Thus, protecting healthcare providers is an important action in the framework of the public health strategies to deal with the pandemic. Short and effective interventions should guarantee the mental well-being of healthcare workers exposed to COVID-19 [41]. In addition, some authors [42] have recently studied the concept of moral injury in the health and emergency service organizations during the current COVID-19 pandemic. As described by Litz, moral injury reflects the psychosocial, behavioral, and even spiritual impacts of “failing to prevent, or bearing witness to acts that transgress deeply held moral beliefs and expectations” [43].

Researchers in this field have highlighted the potential for moral injury amongst healthcare workers faced with systemic and situational barriers to treating patients with limited resources guaranteed by their employers and government. Thus, research into the psychological impact on healthcare workers during the COVID-19 pandemic should take into account this important phenomenon, in order to better manage the risks and mitigate moral distress in those exposed to potentially morally injurious events in the course of their work. The findings are still limited in this field and researchers have been identifying whether and how peer support interventions respond to instances of moral distress and moral injury, in order to protect service workers.

## Figures and Tables

**Table 1:** Socio-demographic characteristics of healthcare professionals.

Socio-Demographic Characteristics	*n*	*%*
Gender		
Female	218	80.2
Male	54	19.8
Age, years, mean (SD)	47	10
Education Level		
Middle school	23	8.4
High school	65	23.8
University	137	50.2
Medical specialization	48	17.6
Marital Status		
Married	138	50.5
Cohabitant	50	18.3
Engaged	22	8.1
Divorced	18	6.6
Single	41	15.0
Widower/widow	4	1.5
Children		
Yes	174	63.3
No	101	36.7
Number of children		
1	86	50.0
2	62	36.0
3	17	9.9
more than 3	7	4.1
Far from the family		
Yes	94	34.2
No	181	65.8
Type employment in the hospital		
Administrative area	21	7.6
Technical area	8	2.9
Health area	246	89.5

**Table 2:** Description of the sample stratified according to Stress, Depression, and Anxiety scales.

		PSS			PHQ-9			GAD-7		
Variable	Category	PSS < 25 (*n* = 193)	PSS ≥ 25 (*n* = 82)	*p*-Value	PHQ-9 < 15 (*n* = 212)	PHQ-9 ≥ 15 (*n* = 63)	*p*-Value	GAD-7 < 15 (*n* = 228)	GAD-7 ≥ 15 (*n* = 47)	*p*-Value
Area, *n* (%)	Health	170 (88.1)	76 (92.7)	0.291	187 (88.2)	59 (93.6)	0.252	201 (88.2)	45 (95.7)	0.189
	Administrative	16 (8.3)	5 (6.1)	0.627	18 (8.5)	3 (4.8)	0.425	19 (8.3)	2 (4.3)	0.546
	Technical	7 (3.6)	1 (1.2)	0.443	7 (3.3)	1 (1.6)	0.687	8 (3.5)	0 (0)	0.359
Gender, *n* (%)	Female	147 (76.6)	71 (88.3)	0.022 *	166 (79.1)	52 (83.9)	0.403	180 (79.6)	38 (82.6)	0.839
	Male	45 (23.4)	9 (11.3)		44 (20.9)	10 (16.1)		46 (20.4)	8 (17.4)	
Age, years	Median (IQR)	50 (39–55)	47 (38–52)	0.103	49 (39–55)	48 (38–54)	0.572	48.5 (39–55)	48 (38–53)	0.791
	(min–max)	(23–66)	(26–67)		(24–66)	(23–67)		(23–66)	(26–67)	
Age classes, years	<45	71 (36.8)	32 (39)	0.726	80 (37.7)	23 (36.5)	0.860	87 (38.2)	16 (34)	0.596
	45–55	67 (34.7)	37 (45.1)	0.104	77 (36.3)	27 (42.9)	0.348	82 (35.9)	22 (46.8)	0.163
	>55	55 (28.5)	13 (15.9)	0.026 *	55 (25.9)	13 (20.6)	0.391	59 (25.9)	9 (19.2)	0.330
Education level, *n* (%)	Middle school	10 (5.2)	13 (16.1)	0.007 *	17 (8.1)	6 (9.5)	0.795	19 (8.4)	4 (8.5)	0.981
	High school	41 (21.3)	24 (29.6)	0.152	42 (20.0)	23 (36.5)	0.007 *	49 (21.7)	16 (34)	0.070
	University	101 (52.6)	36 (44.4)	0.201	110 (52.4)	27 (42.9)	0.185	115 (50.9)	22 (46.8)	0.611
	Medical specialization	40 (20.8)	8 (9.9)	0.028 *	41 (19.5)	7 (11.1)	0.131	43 (19.0)	5 (10.6)	0.209
Marital status, *n* (%)	Married	103 (53.6)	35 (43.2)	0.105	102 (48.6)	36 (57.1)	0.233	113 (49.8)	25 (54.4)	0.670
	Cohabitant	30 (15.6)	20 (24.7)	0.089	42 (20.0)	8 (12.7)	0.264	44 (19.4)	6 (13.0)	0.406
	Engaged	19 (9.9)	3 (3.7)	0.094	20 (9.5)	2 (3.2)	0.120	19 (8.4)	3 (6.5)	0.999
	Divorced	11 (5.7)	7 (8.6)	0.427	14 (6.7)	4 (6.4)	0.999	13 (5.7)	5 (10.9)	0.191
	Single	26 (13.5)	15 (18.5)	0.355	29 (13.8)	12 (19.1)	0.318	34 (15.0)	7 (15.2)	0.999
	Widower/widow	3 (1.6)	1 (1.2)	0.999	3 (1.4)	1 (1.6)	0.999	4 (1.8)	0 (0)	0.999
Children, *n* (%)	None	74 (38.5)	27 (33.3)	0.394	77 (36.7)	24 (38.1)	0.798	85 (37.6)	16 (34.0)	0.675
	1	52 (27.1)	34 (42.0)	0.018 *	67 (31.9)	19 (30.2)	0.828	70 (31.0)	16 (34.0)	0.653
	2 or more	66 (34.4)	20 (24.7)	0.109	66 (31.4)	20 (31.7)	0.926	71 (31.4)	15 (32.0)	0.917
Far from family, *n* (%)	Yes	57 (29.5)	37 (45.1)	0.013 *	68 (32.1)	26 (41.3)	0.177	82 (32.3)	22 (41.5)	0.197
	No	136 (70.5)	45 (54.9)		144 (67.9)	37 (58.7)		172 (67.7)	31 (58.5)	

PSS: Perceived Stress Scale; PHQ-9: Patient Health Questionnaire; GAD-7: General Anxiety Disorder. Continuous variables are described as the mean and standard deviation (SD) or median and interquartile range (IQR). * Two-tailed *p*-value < 0.05 (significant).

**Table 3:** Multivariate logistic regression, according to Stress, Depression, and Anxiety scales.

		PSS			PHQ-9			GAD-7		
Variables	Characteristics	OR	CI 95% for OR	*p*-Value	OR	CI 95% for OR	*p*-Value	OR	CI 95% for OR	*p*-Value
**Area**										
(ref. administrative/technical)	Health	1.93	0.69–5.38	0.207	2.15	0.69–6.70	0.183	3.11	0.70–13.94	0.137
**Gender**										
(ref. male)	Female	1.84	0.79–4.24	0.153	1.09	0.48–2.45	0.833	0.88	0.36–2.15	0.779
**Age**										
(ref. < 45)	45–54	0.43	0.19–0.99	0.050 *	0.68	0.29–1.60	0.382	1.28	0.51–3.23	0.599
	≥55	0.23	0.09–0.59	0.002 *	0.47	0.19–1.22	0.123	0.67	0.24–1.93	0.468
**Education Level**										
(ref. high school or less)	University/medical specialization	0.25	0.12–0.53	<0.001 *	0.34	0.16–0.71	0.004 *	0.62	0.28–1.38	0.243
**Marital Status**										
(ref. married/engaged/cohabitant)	Single/divorced/widower/widow	1.57	0.77–3.24	0.213	1.40	0.67–2.93	0.365	1.29	0.57–2.94	0.542
**Children**										
(ref. none)	1	3.05	1.40–6.64	0.005 *	1.25	0.56–2.79	0.583	1.68	0.69–4.11	0.254
	2 or more	1.51	0.65–3.48	0.333	1.38	0.6–3.17	0.448	1.34	0.52–3.46	0.543
**Far from family**										
(ref. no)	Yes	2.24	1.18–4.20	0.012 *	1.51	0.79–2.88	0.207	1.71	0.83–3.48	0.144

PSS: Perceived Stress Scale; PHQ-9: Patient Health Questionnaire; GAD-7: General Anxiety Disorder; CI: Confidence Interval; OR: Odds Ratio; ref.: reference category; * *p*-value < 0.05.

**Table 4:** Multivariate logistic regression, according to Stress, Depression, and Anxiety scales, stratified by gender.

		PSS				PHQ-9				GAD-7			
		Female	Male			Female		Male		Female		Male	
Variables	Characteristics	OR (CI 95%)	*p*-Value	OR (CI 95%)	*p*-Value	OR (CI 95%)	*p*-Value	OR (CI 95%)	*p*-Value	OR (CI 95%)	*p*-Value	OR (CI 95%)	*p*-Value
**Area**													
(ref. administrative/technical)	Health	1.98 (0.65–6.00)	0.226	8.43 (0.2–34.9)	0.262	2.40 (0.66–8.79)	0.186	0.98 (0.07–14.3)	0.994	5.09 (0.65–39.6)	0.120	0.33 (0.01–7.01)	0.483
**Age, years**													
(ref < 45)	45–54	0.46 (0.19–1.08)	0.076	0.51 (0.02–10.6)	0.666	0.65 (0.25–1.67)	0.377	0.61 (0.04–9.06)	0.723	1.45 (0.53–40.2)	0.468	-	-
	≥55	0.27 (0.09–0.73)	0.010 *	0.05 (0.01–1.82)	0.102	0.40 (0.13–1.22)	0.108	0.96 (0.12–7.50)	0.969	0.58 (0.16–2.10)	0.408	2.66 (0.21–32.8)	0.447
**Education Level**													
(ref. high school or less)	University/medical specialization	0.29 (0.14–0.63)	0.002 *	0.04 (0.01–1.12)	0.059	0.27 (0.12–0.63)	0.002 *	1.72 (0.13–22.5)	0.679	0.60 (0.26–1.44)	0.258	1.10 (0.07–16.1)	0.943
**Marital Status**													
(ref. married/engaged/cohabitant)	Single/divorced/widower-widow	1.58 (0.74–3.37)	0.237	1.90 (0.18–20.2)	0.594	1.39 (0.62–3.11)	0.421	1.37 (0.15–12.1)	0.778	1.28 (0.50–3.12)	0.628	1.45 (0.15–13.6)	0.746
**Children**													
(ref. none)	1	2.82 (1.24–6.40)	0.013 *	32.1 (0.88–12.8)	0.058	1.04 (0.43–2.50)	0.928	3.81 (0.39–37.2)	0.250	1.76 (0.65–4.79)	0.264	1.05 (0.07–15.9)	0.973
	2 or more	1.60 (0.66–3.89)	0.297	0.37 (0.01–11.2)	0.575	1.37 (0.55–3.43)	0.496	0.94 (0.09–9.22)	0.956	1.69 (0.59–4.91)	0.329	0.29 (0.01–5.67)	0.412
**Far from the family**													
(ref. no)	Yes	1.99 (1.01–3.91)	0.045 *	15.31 (0.7–323.8)	0.080	1.39 (0.67–2.86)	0.370	4.41 (0.72–26.9)	0.108	1.59 (0.71–3.58)	0.255	6.17 (0.77–49.5)	0.087

PSS: Perceived Stress Scale; PHQ-9: Patient Health Questionnaire; GAD-7: General Anxiety Disorder; CI: Confidence Interval; OR: Odds Ratio; ref.: reference category; * *p*-value < 0.05.

## Data Availability

The data that support the findings of this study are available from the corresponding author, upon reasonable request.
